# NiS_2_/NiS/Mn_2_O_3_ Nanofibers with Enhanced Oxygen Evolution Reaction Activity

**DOI:** 10.3390/molecules29163892

**Published:** 2024-08-17

**Authors:** Bin Yang, Xinyao Ding, Lifeng Feng, Mingyi Zhang

**Affiliations:** Key Laboratory for Photonic and Electronic Bandgap Materials, Ministry of Education, School of Physics and Electronic Engineering, Harbin Normal University, Harbin 150025, China

**Keywords:** NiS_2_, Mn_2_O_3_, OER, nanofibers, electrospinning

## Abstract

The development of efficient and cost-effective electrocatalysts is crucial for achieving a green hydrogen economy through electrocatalytic water splitting. Herein, we report an excellent catalyst, one-dimensional NiS_2_/NiS/Mn_2_O_3_ nanofibers prepared by electrospinning, which exhibits outstanding electrochemical performance in an alkaline solution. We explored effective strategies to construct one-dimensional nanostructures and composite oxides to promote the electrocatalytic performance of transition metal dichalcogenides. At a current density of 20 mA cm^−2^, it requires an overpotential of 333 mV for OER. Furthermore, NiS_2_/NiS/Mn_2_O_3_ nanofibers maintain good durability even after 1000 cycles. The long-term electrochemical stability test of the catalyst NiS_2_/NiS/Mn_2_O_3_ was implemented at 20 mA cm^−2^ for 12 h. The potential remained at 99.52%. Therefore, this study demonstrates that NiS_2_/NiS/Mn_2_O_3_ can serve as a viable green hydrogen production electrocatalyst.

## 1. Introduction

Recently, there has been a growing recognition that electrochemical water splitting is a feasible method for generating clean, efficient, and sustainable hydrogen energy [1,2,3,4]. Water electrolysis comprises two primary reactions: the cathodic Hydrogen Evolution Reaction (HER) and the anodic Oxygen Evolution Reaction (OER) [5]. OER is one of the two half-reactions involved in water electrolysis. It involves the formation of two oxygen-oxygen bonds and the transfer of multiple protons and electrons, which substantially impact the reaction kinetics and catalytic efficiency. Efficient electrocatalysts are essential to accelerate OER. It is widely recognized that precious metal materials, such as RuO_2_ and IrO_2_, display high activity for OER [6]. However, their use is limited by the high cost and the scarcity of precious metal elements in nature. Therefore, the essential objective of research and development on Earth is to investigate transition metal-based electrocatalysts that are abundant, cost-effective, and high-performing [7,8,9,10].

Recently, various cost-effective and readily available materials have been developed as alternatives for OER catalysts. These materials include transition metal (Mn, Fe, Co, Ni, Cu, and so on) oxides [11], sulfides [12], selenides [13], nitrides [14], carbides [15], and more. Among these, chalcogenide compounds, especially MSx, have a promising future due to their excellent performance, stability, and ease of preparation, making them attractive for a wide range of applications [16]. As an important half-reaction for electrocatalytic water splitting, it requires high energy to drive the occurrence of OER due to the complex four-electron transfer process and slow O-O bond formation steps involved. Electrocatalysts can effectively reduce the reaction overpotential and increase the reaction activity, and their performance is related to the number of active sites and the intrinsic catalytic activity. Therefore, the OER reaction performance can be further improved by the controllable preparation of materials with high intrinsic activity and the exposure of more active areas. However, these electrocatalysts often fall short of meeting commercial requirements due to their relatively slow charge transfer abilities and limited exposure to active sites. Structural engineering is an effective approach to augment the electrochemically active area and expose a greater number of active sites.

Various nanostructures have been designed to improve the electrocatalytic performance of chalcogenide compounds, including nanoparticles [17], nanosheets [1,18], nanowires [19], and nanofibers. One-dimensional nanofiber structures have gained considerable attention recently due to their relatively high specific surface area, plentiful active sites, and shorter electron transfer pathways [20,21]. Catalysts with one-dimensional structures possess a substantial surface area, enabling them to adsorb a variety of compounds [22]. Electrospinning technology is considered a straightforward, universal, and economical method for large-scale preparation of one-dimensional nanomaterials. One-dimensional fiber materials produced through electrospinning offer advantages such as a large specific surface area, tunable chemical composition, morphology, fiber diameter, and high porosity. These features make them highly promising for a wide range of applications in the field of catalysis [23]. Here, we prepared NiS_2_/NiS/Mn_2_O_3_ nanofibers using techniques such as electrospinning, thermal annealing, and sulfurization. Due to the unique one-dimensional structure of NiS_2_/NiS/Mn_2_O_3_ nanofibers, they exhibit improved charge/transport properties and a multitude of electrocatalytic active sites. The stacked nanoparticles construct NiS_2_/NiS/Mn_2_O_3_ nanofibers with tough surfaces to expose abundant interfacial active sites, and the one-dimensional nanostructures facilitate unhindered charge transport, which accelerates the introduction of catalytic kinetics Mn to adjust the structure of electrons NiS_2_/NiS, which greatly improves the performance of OER. NiS_2_/NiS/Mn_2_O_3_ nanofibers exhibit excellent electrocatalytic activity in an alkaline electrolyte, driving a current density of 20 mA cm^−2^ with a minimal overpotential of 333 mV. Furthermore, NiS_2_/NiS/Mn_2_O_3_ nanofibers maintain good durability even after 1000 cycles. The NiS_2_/NiS/Mn_2_O_3_ catalyst, with its distinctive one-dimensional structure, holds the promise of developing into a potential substitute for precious metal-based OER catalysts.

## 2. Result and Discussion

**Figure 1:**
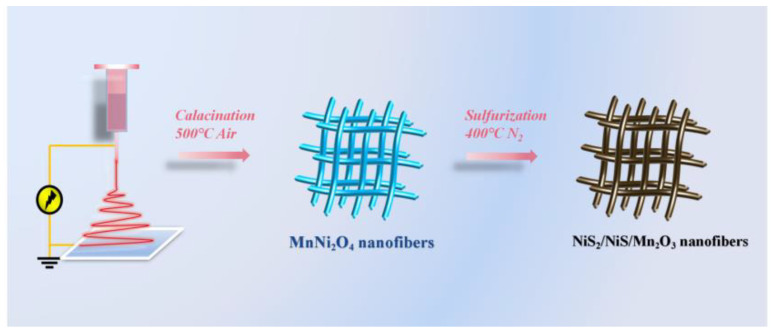
Schematic illustration of the fabrication of NiS_2_/NiS/Mn_2_O_3_ nanofibers.

Figure 1 illustrates the synthesis process of NiS_2_/NiS/Mn_2_O_3_ nanofibers. Initially, MnNi_2_O_4_ nanofibers were prepared through electrospinning. Subsequently, NiS_2_/NiS/Mn_2_O_3_ nanofibers were synthesized via a tube furnace sulfurization process, during which the preformed MnNi_2_O_4_ nanofibers underwent a reaction with H_2_S gas produced by the thermal decomposition of sulfur powder.

**Figure 2:**
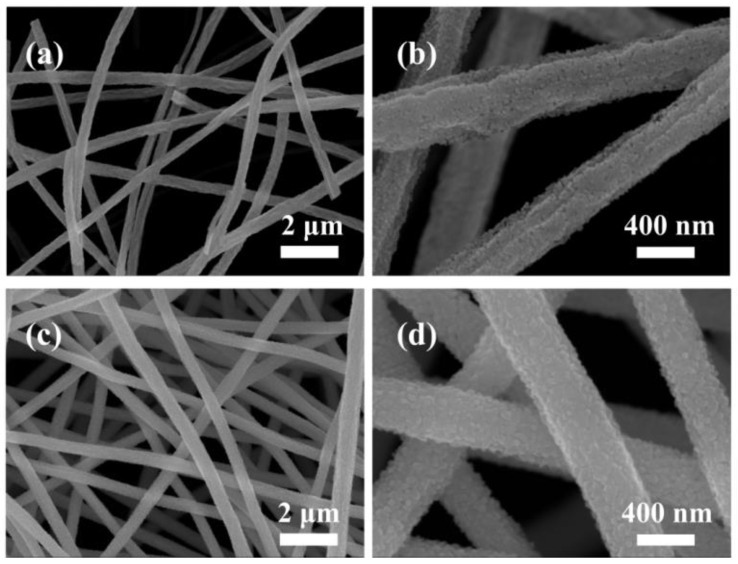
The SEM images of MnNi_2_O_4_ nanofibers (**a**,**b**), NiS_2_/NiS/Mn_2_O_3_ nanofibers (**c**,**d**).

The microstructure and morphological details of the as-prepared NiS_2_/NiS/Mn_2_O_3_ nanofibers were initially observed using SEM. Upon high-temperature calcination, MnNi_2_O_4_ nanoparticles self-assemble in an orderly fashion, forming a one-dimensional nanostructure. Upon closer examination, it becomes evident that the fiber’s diameter is approximately 500 nm (Figure 2a,b). After the sulfurization treatment, the microstructural characteristics of NiS_2_/NiS/Mn_2_O_3_ nanofibrous materials remained mostly unchanged. It was evident that the nano-crystalline grains were intricately stacked, creating one-dimensional nanofibrous structures. As illustrated in Figure 2c,d, it is clear that NiS_2_/NiS/Mn_2_O_3_ nanofibers, characterized by their one-dimensional structure, display a substantially increased specific surface area. This unique structural trait enhances stability and promotes electron transport, ultimately leading to an enhancement of electrocatalytic activity.

**Figure 3:**
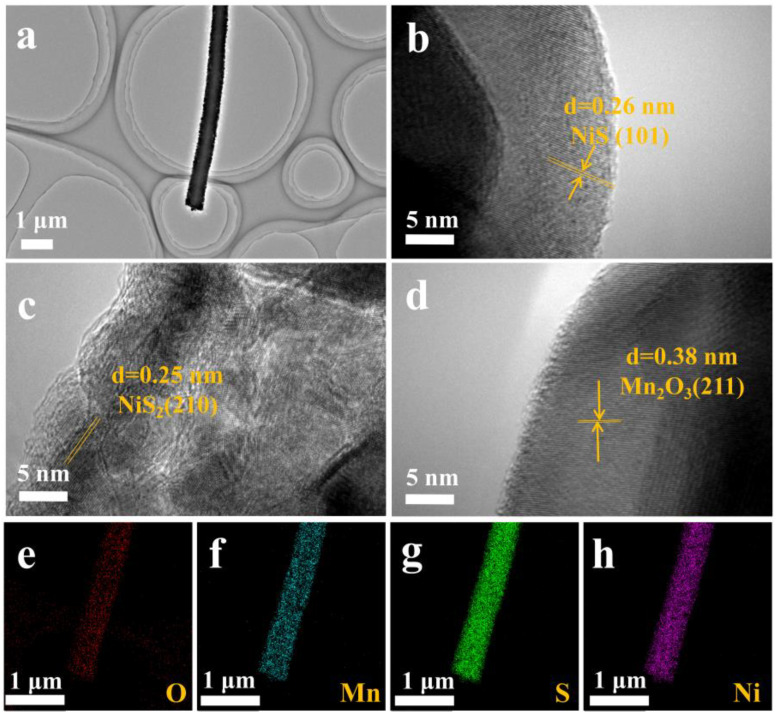
TEM of MnNi_2_O_4_ nanofibers (**a**), HRTEM images of MnNi_2_O_4_ nanofibers (**b**–**d**). The corresponding elemental mappings of O, Mn, S and Ni of MnNi_2_O_4_ nanofibers (**e**–**h**).

The one-dimensional structure of MnNi_2_O_4_ and NiS_2_/NiS/Mn_2_O_3_ nanofibers was further analyzed through Transmission Electron Microscopy (TEM). The clear structure of MnNi_2_O_4_ nanofibers is readily apparent in Figure 3a, consistent with what is observed under SEM. The high-resolution TEM (HRTEM) image in Figure 3a distinctly reveals lattice fringes with an interplanar spacing of 0.25 nm, closely corresponding to the d-spacing of the MnNi_2_O_4_ (311) plane. Figure 3c–f are examined using spectral mapping, revealing the uniform distribution of Ni, Mn, and O elements. Figure 3a showcases TEM images of NiS_2_/NiS/Mn_2_O_3_. It is evident that NiS_2_/NiS/Mn_2_O_3_ retains the same morphology as MnNi_2_O_4_ before vulcanization, and the vulcanization process does not disrupt the one-dimensional structure. This exceptional nanoscale architecture fosters interactions between grains and expedites electron transfer. The corresponding high-resolution TEM images can be found in Figure 3b–d. In these images, lattice spacings of 0.26 nm, 0.25 nm, and 0.38 nm correspond to the (101) crystal plane of NiS, the (210) crystal plane of NiS_2_, and the (211) crystal plane of Mn_2_O_3_, respectively. Moreover, elemental mapping images clearly demonstrate the distribution of O, Mn, S, and Ni elements throughout the entire sample area (Figure 3e–h).

**Figure 4:**
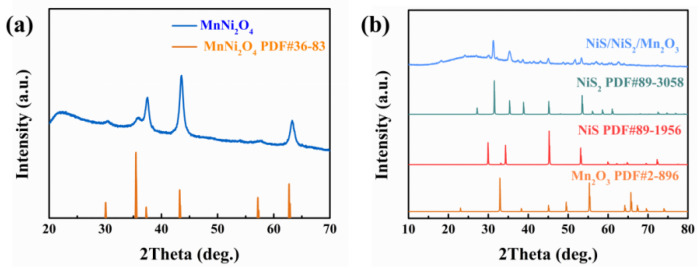
The XRD patterns of (**a**) MnNi_2_O_4_ nanofibers and (**b**) NiS_2_/NiS/Mn_2_O_3_ nanofibers.

The crystal structure of MnNi_2_O_4_ was analyzed using X-ray diffraction (XRD) and NiS_2_/NiS/Mn_2_O_3_ nanofibers, as depicted in Figure 4. The XRD pattern (Figure 4a) of MnNi_2_O_4_ nanofibers closely matches the diffraction peaks of the spinel structure MnNi_2_O_4_ (PDF#36-83). In Figure 4b, after the sulfurization of MnNi_2_O_4_ nanofibers, two distinct sulfides (NiS_2_ and NiS) are obtained, and their characteristic diffraction peaks perfectly match those of NiS_2_ (PDF#89-3058) and NiS (PDF#89-1958), respectively. The characteristic peaks of NiS_2_/NiS/Mn_2_O_3_ nanofibers at 31.4°, 35.19°, 38.6°, 45.25°, and 53.5° correspond to the orthorhombic crystal planes of NiS_2_ (PDF#89-3058) (200), (210), (211), (220), and (311). Additionally, the characteristic peaks of NiS_2_/NiS/Mn_2_O_3_ nanofibers at 29.7°, 34.2°, 45.1°, and 53.2° match the orthorhombic crystal planes of NiS (PDF#89-1956) (100), (101), (102), and (110), respectively. Notably, the XRD pattern also reveals a distinctive peak at 49.4°, which accords with the (431) crystal plane of Mn_2_O_3_ (PDF#2-896). This further confirms the successful synthesis of NiS_2_/NiS/Mn_2_O_3_ nanofibers.

**Figure 5:**
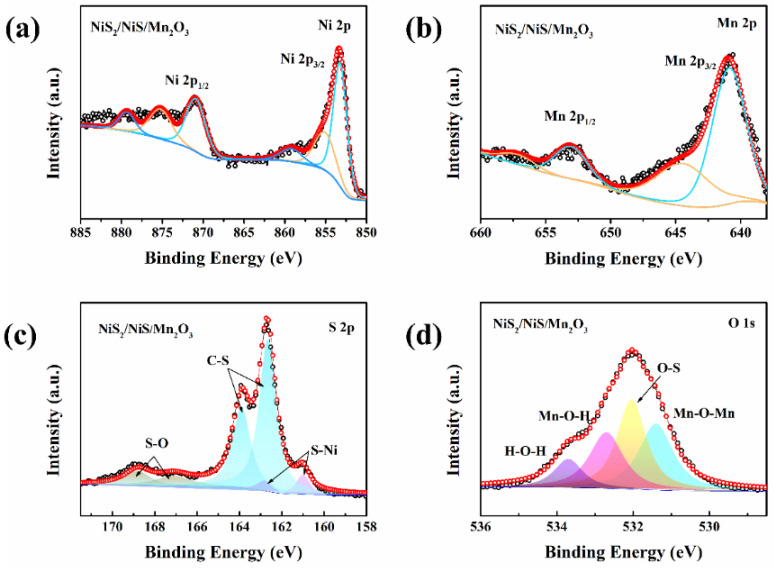
The high-resolution XPS spectra of (**a**) Ni 2p, (**b**) S 2p, (**c**) Mn 2p, and (**d**) O 1s of NiS_2_/NiS/Mn_2_O_3_ nanofibers.

The Ni 2p spectrum of the NiS_2_/NiS/Mn_2_O_3_ catalyst can be resolved into six peaks (Figure 5a) [24]. The peak at 872.1 eV is assigned to Ni 2p_1/2_, while the satellite peaks labeled “Sat” correspond to 859.2 and 879.3 eV. From Figure 5b, it can be observed that there are four distinct peaks for element Mn 2p [25]. Specifically, the peaks at 640.9 and 644.4 eV are assigned to Mn 2p_3/2_, while the peaks at 653.1 and 657.1 eV are allocated to Mn 2p_1/2_. In Figure 5c, the peaks at 160.8 and 162.8 eV are attributed to Ni-S bonds, signifying their involvement in the formation of NiS and NiS_2_. The presence of S-C bonding could be a consequence of the interaction between S and C elements during sample synthesis. A pair of less pronounced peaks at 167.3 and 168.9 eV indicate the presence of a typical S-O bond, indicating a low degree of sulfur oxidation. Figure 5d shows the O 1s electron peak, in which a peak at 531.2 eV is attributed to Mn-O-Mn, mainly derived from Mn_2_O_3_ in the NiS_2_/NiS/Mn_2_O_3_ catalyst. These results confirmed the successful synthesis of the NiS_2_/NiS/Mn_2_O_3_ catalyst.

**Figure 6:**
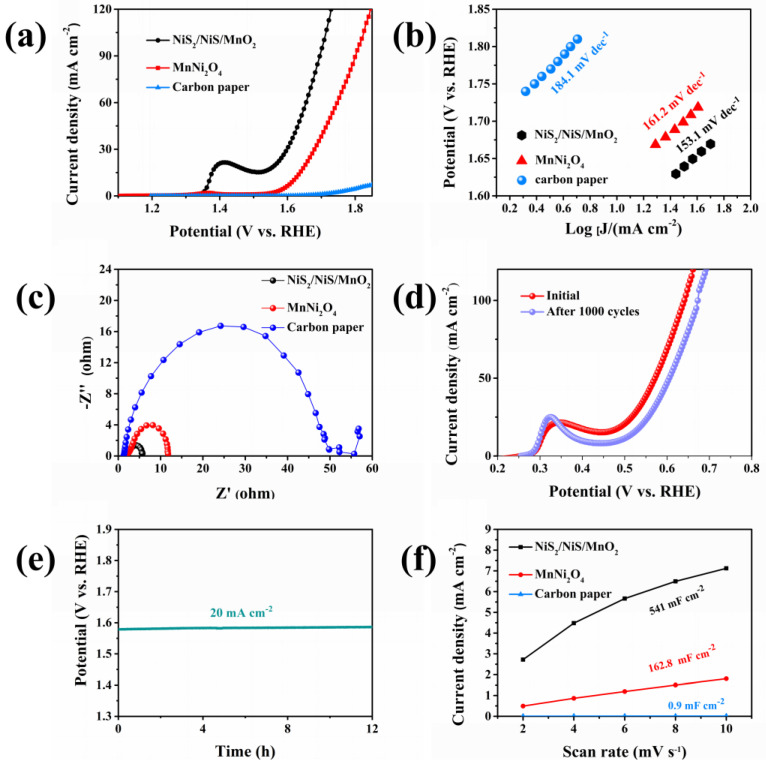
Electrocatalytic OER performance of NiS_2_/NiS/Mn_2_O_3_, MnNi_2_O_4_ and carbon paper. (**a**) LSV curves of the synthesized electrodes. (**b**) Corresponding Tafel plots of the above catalysts. (**c**) *EIS* pattern of the above catalysts investigated at a constant potential of 0.5 V (vs. SCE). (**d**) CV curves before and after 1000 cycles. (**e**) Long-term stability of NiS_2_/NiS/Mn_2_O_3_ at the current density of 20 mA cm^−2^ for 12 h. (**f**) ∆J/2 as a function of scan rate of NiS_2_/NiS/Mn_2_O_3_, MnNi_2_O_4_, Carbon paper.

The oxygen evolution performance of the one-dimensional NiS_2_/NiS/Mn_2_O_3_ nanofibers was assessed using a three-electrode system in a 1 M KOH electrolyte. Additionally, the electrochemical performance of MnNi_2_O_4_ and carbon paper was examined under identical conditions for comparison. In Figure 6a, we observe that the low OER activity of the carbon paper can be neglected, and the results show that the catalytic activity for the reaction is entirely attributed to our samples. The LSV curves presented reveal that NiS_2_/NiS/Mn_2_O_3_ nanofibers exhibit the lowest onset potential and deliver the highest current density. The overpotential for NiS_2_/NiS/Mn_2_O_3_ nanofibers at a current density of 20 mA cm^−2^ is 333 mV, which is lower than that of MnNi_2_O_4_ (420 mV). These results demonstrate that the NiS_2_/NiS/Mn_2_O_3_ nanofibers exhibit the highest OER activity. At the same time, we compared the electrochemical performance of the catalyst associated with NiS_2_/NiS/Mn_2_O_3_ [26,27,28,29,30,31,32,33], and it is evident that NiS_2_/NiS/Mn_2_O_3_ exhibits excellent electrochemical performance (Table S1). The Tafel slope is a key parameter for evaluating the catalytic activity of electrocatalysts in water splitting. As depicted in Figure 6b, the Tafel slope of NiS_2_/NiS/Mn_2_O_3_ nanofibers is relatively small, measuring 153.1 mV dec^−1^. This Tafel slope is lower than that of MnNi_2_O_4_ (161.2 mV dec^−1^) and carbon paper (184.1 mV dec^−1^). A lower Tafel slope indicates that NiS_2_/NiS/Mn_2_O_3_ nanofibers exhibit faster kinetic response, further substantiating their effectiveness as a catalyst.

Oxygen evolution reaction (OER) is a 4-electron complex process that takes place at the anode in an electrolytic cell. The OER process is considered to be a slower kinetic process in which three absorbing intermediates are generated: M-OOH_ads_, M-OH_ads_, and M-OH_ads_. The OER process takes the dissociation of H_2_O in the acidic electrolyte or the coordination of OH in the basic electrolyte as the first step. The next immediate steps involved the oxidation of M-OH_ads_ to M-OH_ads_, oxidation of M-OH_ads_ to M-OOH_ads_, and oxidation of M-OOH_ads_ to O_2_. According to many studies reported, the appropriate OER mechanism in alkaline electrolytes is as follows:

M + OH^−^ → MOH_ads_ + e^−^ (1)

MOH_ads_ + OH^−^ → MO_ads_ + H_2_O + e^−^ (2)

MO_ads_ + MO_ads_ → 2M + O_2_ (3)

or MO_ads_ + OH^−^ → MOOH_ads_ + e^−^ (4)

MOOH_ads_ + OH^−^ → M + O_2_ + H_2_O + e^−^ (5)

where M is the active site on the catalyst surface and “ads” is the type of adsorption on the catalyst surface. Two major pathways affect oxygen production through different fundamental steps. The first step is the direct binding of two M-O_ads_ intermediates following the steps of (1) → (2) → (3). The second step follows the sequence (1) → (2) → (4) → (5). First, M-O_ads_ are coupled to OH^−^ to form the intermediate M-OOH_ads_, and then combined with another OH^−^ to form O_2_. It is worth mentioning that the thermodynamic barrier of reaction (3) is always larger than that of reactions (4) and (5) [34].

To gain a deeper understanding of the electrocatalytic efficiency of these catalysts for water oxidation, we conducted electronic conductivity measurements at a 500 mV overpotential using Electrochemical Impedance Spectroscopy (EIS). In Figure 6c, the low-range Nyquist plot reveals various charge transfer resistances (*R*_ct_) represented by the semicircle. This observation indicates that the variations in *R*_ct_ for these electrocatalysts align with the trend in water oxidation activity as indicated by the polarization curves. The images indicate that NiS_2_/NiS/Mn_2_O_3_ nanofibers exhibit the lowest resistance, implying that, among these catalysts, NiS_2_/NiS/Mn_2_O_3_ nanofiber electrodes facilitate faster electron transport between the electrode and the electrolyte, thus demonstrating superior catalytic performance. To evaluate the stability of NiS_2_/NiS/Mn_2_O_3_, we performed a cyclic voltammetry (CV) scanning test over 1000 cycles in a 1M KOH solution. As shown in Figure 6d, both the LSV curve and the chronopotential show minimal changes after 1000 cycles, demonstrating excellent durability. In addition, the NiS_2_/NiS/Mn_2_O_3_ was tested for long-term electrochemical stability at 20 mA cm^−2^ for 12 h, and the potential was maintained at 99.52% after the electrochemical stability test, showing excellent durability (Figure 6e). To elucidate the high electrochemical activity of NiS_2_/NiS/Mn_2_O_3_, we measured the double-layer capacitance (*C*_dl_) of the electrode using the cyclic voltammetry (CV) method at various scan rates (2, 4, 6, 8, and 10 mV s^−1^) to evaluate the electrochemical surface area (ECSA) of the material in the non-Faradaic region (Figure S1). The CV curves are depicted in Figure 6f. it is evident that the *C*_dl_ of NiS_2_/NiS/Mn_2_O_3_ nanofibers is 541 mF cm^−2^, which is significantly superior to that of MnNi_2_O_4_ (161.2 mF cm^−2^) and carbon paper (0.9 mF cm^−2^). This indicates that NiS_2_/NiS/Mn_2_O_3_ nanofibers possess a larger electrochemical surface area, therefore providing a substantial number of exposed active sites to facilitate the electrochemical reaction process.

## 3. Experimental Section

### 3.1. Materials

Sigma-Aldrich (St. Louis, MO, USA) provided the polyacrylonitrile (PAN, Mw~150,000). Mn(CH_3_COO)_2_·4H_2_O, Ni(CH_3_COO)_2_·6H_2_O, and N, N-dimethylformamide (DMF) were obtained from Zhiyuan Reagent (Tianjin, China). Aladdin provided and sublimated sulfur. All experimental drugs were analytically pure and performed without further processing.

### 3.2. Synthesis of MnNi_2_O_4_ Nanofibers

In a typical preparation process, 0.5 g of PAN was dissolved in 5 mL of DMF solution and stirred until a clear solution was obtained. A total of 0.34 g Mn(CH_3_COO)_2_·4H_2_O and 0.18 g Ni(CH_3_COO)_2_·6H_2_O were sequentially added to the clear solution, followed by stirring for 12 h until well blended. After adding the mixed precursor solution to the syringe, adjust the working voltage to approximately 7 kV and maintain a collection distance of 15 cm. Fiber membranes were collected following 12 h of electrospinning. To obtain MnNi_2_O_4_ nanofibers, incinerate the nanofibers at 500 °C in an air atmosphere for 2 h with a heating rate of 2 °C per minute.

### 3.3. Synthesis of NiS_2_/NiS/Mn_2_O_3_ Nanofibers

For the synthesis of NiS_2_/NiS/Mn_2_O_3_ nanofibers, 20 mg of MnNi_2_O_4_ nanofibers and 400 mg of sulfur powder were positioned at both ends inside a nitrogen-filled tube furnace. The sulfur powder is positioned on the upstream air inlet side, while the samples are positioned at a distance from a single porcelain boat. Subsequently, heat the tube furnace to 400 °C in an air atmosphere at a rate of 2 °C per minute and maintain this temperature for 2 h. Following natural cooling, black NiS_2_/NiS/Mn_2_O_3_ nanofibers were successfully obtained.

### 3.4. Materials Characterization

The crystal structure was determined through the analysis of X-ray diffraction patterns (XRD, D/max 2600, Rigaku, Tokyo, Japan). Sample morphology was described using a scanning electron microscope (SEM, SU70, Hitachi, Tokyo, Japan). Transmission electron microscopy (TEM, FEI, Tecnai TF20, Hillsboro, OR, USA) was employed to examine the atomic structure of the catalysts, and the surface chemistry of the samples was studied using X-ray photoelectron spectroscopy (XPS, Thermofisher Scientific Company, Waltham, MA, USA).

### 3.5. Electrochemical Measurements

For the preparation of the working electrode, 20 mg of NiS_2_/NiS/Mn_2_O_3_ nanofibers and 2.5 mg of acetylene black were ground for one hour. Following this, 150 µL of NMP and 50 mL of a 5% Pvdf solution by mass were added separately to the mixed powders and then sonicated for one hour to achieve dispersion. A 20 µL portion of the slurry was then applied to a 1 × 1 cm^2^ carbon paper, and the prepared samples were dried at 60 °C for 12 h. The loading of the catalyst on the carbon paper is 2 mg.

The electrochemical performance of the samples was measured using an electrochemical workstation with a three-electrode system, and the electrolyte used was 1 M KOH solution. Carbon paper, platinum foil, and saturated calomel electrode (SCE) coated with the prepared catalysts were employed as the working, counter, and reference electrodes, respectively. Unless specified otherwise, the potential was converted to the reversible hydrogen electrode (RHE) scale using the equation: E_(RHE)_ = E_(SCE)_ + 0.241 + 0.059 pH. Electrochemical activation of these electrocatalysts was achieved through redox cycling between 0 V and 0.85 V at a scan rate of 100 mV/s. Linear sweep voltammetry (LSV) was performed at a scanning rate of 5 mV/s. Electrochemical impedance spectroscopy (EIS) was conducted across a frequency range spanning from 100 kHz to 0.01 Hz, with an applied potential of 500 mV. Electrochemically active surface area (ECSA) was determined using a double-layer capacitance measurement, a well-established electrochemical technique. Cyclic voltammetry (CV) for the oxygen evolution reaction (OER) was conducted within the range of 1.38–1.48 V (relative to RHE) at scan rates of 2, 4, 6, and so forth, mV/s. Cycling tests were carried out with cyclic voltammetry (CV) for 1000 cycles at a scan rate of 100 mV/s.

## 4. Conclusions

This paper details the preparation of one-dimensional nanostructured NiS_2_/NiS/Mn_2_O_3_ catalysts using electrospinning technology. NiS_2_/NiS/Mn_2_O_3_ benefits from its larger specific surface area, increased electrolyte storage capacity, and minimal electron transfer impedance. In our experiments, one-dimensional NiS_2_/NiS/Mn_2_O_3_ nanofibers exhibit outstanding OER (Oxygen Evolution Reaction) activity. They require only 333 mV overpotential to achieve a current density of 20 mA cm^−2^ and demonstrate excellent stability. This work provides a promising strategy for constructing a durable OER catalyst.

## Data Availability

All the relevant data are included in this published article.

## Supplementary Materials

The following supporting information can be downloaded at: https://www.mdpi.com/article/10.3390/molecules29163892/s1, Figure S1: Cyclic voltammograms in the on non-faradic region for (**a**) NiS_2_/NiS/Mn_2_O_3_, (**b**) MnNi_2_O_4_, (**c**) carbon paper; Table S1: Comparison of the OER performance of NiS_2_/NiS/Mn_2_O_3_ with previous reports. Refs. [35,36,37,38,39,40] are cited in the Supplementary Materials.
