# Supplementary material for: NiS2/NiS/Mn2O3 Nanofibers with Enhanced Oxygen Evolution Reaction Activity
Source: Molecules. 2024 Aug 17;29(16):3892. doi: 10.3390/molecules29163892 (PMC11357288; doi:10.3390/molecules29163892)
Supplement: Supplementary file 1 [file molecules-29-03892-s001.zip › molecules-3096895-supplementary.pdf]

Materials

Polyacrylonitrile (PAN, Mw ~150,000) was provided by Sigma-Aldrich. N,N-dimethylformamide (DMF), $Mn(CH_3COO)_2·4H_2O$, and $Ni(NO_3)_2·6H_2O$ were obtained from Zhiyuan Reagent (Tianjin, China, Alfa Aesar). H_2S was purchased from Aladdin Corporation.

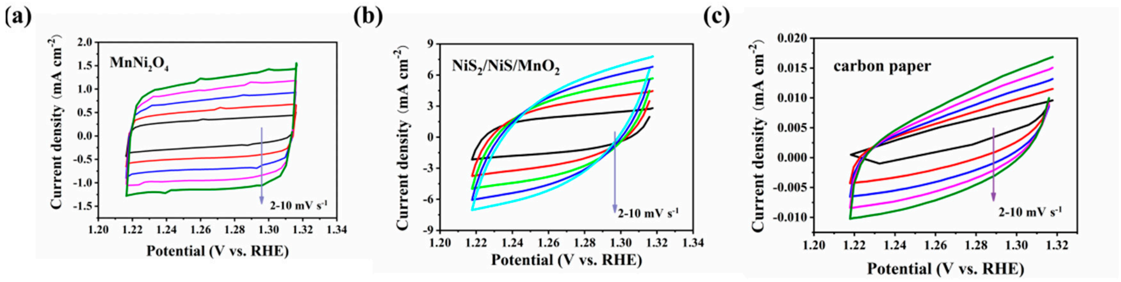

Figure S1. Cyclic voltammograms in the on non-faradic region for (**a**) $NiS_2/NiS/Mn_2O_3$, (**b**) $MnNi_2O_4$, (**c**) carbon paper.

Table S1. Comparison of the OER performance of $NiS_2/NiS/Mn_2O_3$ with previous reports.

Materials	Overpotential	Electrolyte	Stability	Reference
$NiS_2/NiS/Mn_2O_3$	333 mV	1 M KOH	99.52% @20 mA cm^{-2} , 12h	this work
NiS/SPE	362 mV	0.1 M KOH	100% @700 mV, 10h	[1]
NiS_2 @ Ni-B i/CC	486 mV	0.1 M K-Bi	Maintaining its catalytic activity@20 mA cm^{-2} , 24h	[2]
12% Ni-Co_3O_4	340 mV	1 M KOH	None	[3]
NiS/NF	320 mV	1 M KOH	Current density for over 20 hours with a recession of 4.5%@10 mA cm^{-2}	[4]
NiS-Cu_2S-CF	308 mV	1 M KOH	a stable electrocatalyst @ 1.53 V vs. RHE, 30h	[5]
$NiCo_2$ -N/TiN	330 mV	1 M KOH	a constant potential of 1.71 V for 100000 s	[6]
CuO NRs/CF	384 mV	1 M KOH	95.3%@1.6V, 30h	[7]

References

[1] Manjunatha, C.; Srinivasa, N.; Chaitra, S. K. ; Sudeep, M.; Chandra Kumar, R.; Ashoka, S. Controlled synthesis of nickel sulfide polymorphs: studies on the effect of morphology and crystal structure on OER performance. *Materials Today Energy.* **2020**, *12*, 100414.

[2] Ma, X.; Ma, M.; Liu, D.; Hao, S.; Qu, F.; Du, F. ; Asiri, A. M.; Sun, X. Core–Shell-Structured NiS_2@Ni-Bi Nanoarray for Efficient Water Oxidation at Near-Neutral pH. *ChemCatChem.***2017**, *9*, 3138-3143.

[3] Du, H.; Pu, W.; Yang, C.; Morphology control of Co_3O_4 with nickel incorporation for highly efficient oxygen evolution reaction. *Applied Surface Science*. **2021**, *541*, 148221.

[4] Ren, J.; Yuan, Z. Hierarchical Nickel Sulfide Nanosheets Directly Grown on Ni Foam: A Stable and Efficient Electrocatalyst for Water Reduction and Oxidation in Alkaline Medium. *ACS Sustainable Chemistry & Engineering*. **2017**, *5,* 7203–7210.

[5] Wang, L,; Li, M. C.; Lyu ,Y. X.; Liu, J. W.; Du, J. M. Cu_2S Nanoflakes Decorated with NiS Nanoneedles for Enhanced Oxygen Evolution Activity. *Micromachines.* **2022**, 13(2), 278.

[6] Tan, C. T.; Wang, F. H.; Lv, K.; Shi, Y. Y; Dong, B. B; Hao, L. Y.; Yin, L. J.; Xu, X.; Xian, Y. X.; Agathopoulos, X. TiN ceramic membrane supported nitrogen-incorporating $NiCo_2$ nanowires as bifunctional electrode for overall water splitting in alkaline solution. *Separation and Purification Technology*. **2022**, *298*, 121582.

[7] Nguyen, C.D.; Nguyen, V.; Tuyen, P. N. K.; Pham, L. M. T.; Vu, T. Snowflake Co_3O_4-CuO heteroanode arrays supported on three-dimensional framework for enhanced oxygen evolution. *Journal of Electroanalytical Chemistry*. **2020**, *871*, 114235.
